# Association between TNFα - 308 G/A polymorphism and oral lichen planus (OLP): a meta-analysis

**DOI:** 10.1590/1678-7757-2017-0184

**Published:** 2018-03-26

**Authors:** Yuqiao Zhou, Alexandre Rezende Vieira

**Affiliations:** 1 Sichuan University, West China College of Stomatology, State Key Laboratory of Oral Diseases, Chengdu, China; 2 University of Pittsburgh, School of Dental Medicine, Department of Oral Biology, Pittsburgh, PA, U.S.A

**Keywords:** Oral lichen planus, Tumor necrosis factors, Genetic polymorphism

## Abstract

**Objectives:**

To determine whether Tumor Necrosis Factor alpha (TNFα) –308 G/A polymorphism is associated with oral lichen planus (OLP).

**Material and Methods:**

A systematic electronic search of the literature was conducted to identify all published studies on the association between TNFα –308 G/A polymorphism and OLP. All case-control studies evaluating the TNFα –308 G/A polymorphisms in OLP were selected. A meta-analysis of the studies that fulfilled the inclusion criteria was performed. Odds ratios (OR) with 95% confidence intervals (CI) were also calculated.

**Results:**

Seven studies comprising 450 OLP cases and 867 controls were included in the meta-analysis. In the pooled analysis, TNFα –308 G/A polymorphism was associated with OLP with random effects and OR of 2.33 (95%CI=1.07-5.11; p=0.03), assuming a dominant mode of inheritance (AA+GA vs. GG). In the subgroup analysis by ethnicity, TNFα –308 G/A was associated with a significantly increased odds ratio of OLP in mixed ethnicity (OR=5.22; 95%CI=1.93-14.15; p=0.001), but not in Asians (OR=1.57; 95%CI=0.54-4.54; p=0.41) or Caucasians (OR=1.45; 95%CI=0.19-11.22; p=0.72). For subgroup analysis based on HCV (hepatitis C virus) infection status, significant increased risk of OLP was found among patients with mixed HCV infection status (OR=3.77; 95%CI=1.07-13.2; p=0.038), but not in patients without HCV infection (OR=2.09; 95%CI=0.63-6.91; p=0.22) and patients with HCV infection (OR=0.48; 95%CI=0.13-1.69; p=0.25).

**Conclusion:**

Our results suggest that –308 G/A polymorphism in TNFα is a potential genetic marker for OLP.

## Introduction

Oral lichen planus (OLP) is a chronic inflammatory oral condition characterized by oral lichenoid lesions. The etiopathogenesis of OLP involves an interaction between genetic, environmental, and lifestyle factors. OLP lesions can be induced by drugs or dental materials, and are often called idiopathic, involving abnormal immune responses^16^. Previous studies have suggested the involvement of hepatitis C virus (HCV) infection in the etiology of oral lichen planus^14^. However, the etiopathogenesis of OLP is still unclear.

Cytokines play an important role in the pathogenesis of OLP, and a bulk of evidence suggests that OLP is a T-cell-mediated disease^12^ ^,^ ^16^. TNFα is a potent immunomodulator and proinflammatory cytokine that has been found to play roles in the pathogenesis of multiple inflammatory or autoimmune diseases^2^. A higher level of TNFα has been found in serum from OLP patients compared to controls^15^. The subepithelial T cells of OLP contain TNFα mRNA and express TNFα cytokine^6^ ^,^ ^17^. TNFα production may be partially determined at the genetic level. The TNFα gene is a member of the TNF superfamily located on chromosome 6q21, within the major histocompatibility complex (MHC) class III region^3^. Several polymorphisms and mutations have been identified within TNFα, among which a genetic variation at position –308 of the TNFα gene that results in two allelic forms, including a guanine (G) that represents the common variant and an adenine (A) that defines the less common one. G/A polymorphism at position –308 of the TNFα gene promoter is reported to increase TNFα transcription and produce higher levels of this cytokine^11^ ^,^ ^18^. The possible association between TNFα –308 (rs1800629) G/A polymorphism and OLP has been studied by several investigators^1^ ^,^ ^2^ ^,^ ^5^ ^–^ ^7^ ^,^ ^10^ ^,^ ^19^. However, the results have been inconclusive or even contradictory. Differences in ethnicity, lack of statistical power, and involvement of HCV infections are potential factors underlying the discrepant results of these studies. Meta-analysis is a widely used tool to overcome the problem of the small sample sizes and inadequate statistical power of genetic studies of complex traits^13^. Jin, et al.^9^ (2012) conducted a meta-analysis on TNFα –308 G/A polymorphism and lichen planus and did not find significant association between them. The studies available at that time were limited. Herein, we conducted a meta-analysis to assess the association between genetic variants of TNFα –308 G/A polymorphism and risk of OLP with the most updated literature on this association.

## Material and methods

### Study selection and data extraction

A comprehensive literature search was conducted on Medline, PubMed, Embase, and Science Citation Index for all articles that were published by April 17, 2017 on the association between TNFα –308 G/A polymorphism and OLP risk. The search terms were used as follows: (“oral lichen planus” or “OLP”) and (“tumor necrosis factor” or “TNF” or “tumor necrosis factor-α” or “TNF-α” or “TNF-α” or “tumor necrosis factor-a”) and (“Polymorphism” or “mutation” or “variant”). Selection criteria of an eligible study were: (a) investigation of TNFα gene polymorphism and OLP risk; (b) case-control studies; (c) sufficient genotype distributions for cases and controls so that an OR with 95%CI could be assessed. Two investigators collected the information from all eligible publications based on the abovementioned criteria. Data including publication year, first author, country, ethnicity, HCV infection status, case number and control number, and genotyping method were extracted independently by these two investigators and reached conformity on all items by consultation.

### Statistical study

Odds ratios with 95% confidence intervals were used to estimate the association between the TNFα –308 G/A polymorphism and OLP risk. The pooled ORs were performed assuming a dominant genetic model (AA+AG *vs.* GG). The statistical significance of the ORs was analyzed by Z-test, and p<0.05 was considered statistically significant. In addition to the comparison among all subjects, we also performed subgroup analyses by HCV infection and ethnicity.

Heterogeneity was evaluated by a chi-square-based Q statistic, and statistical significance was assumed for a *p*-value lower than 0.05. A fixed-effect model was used when *p* heterogeneity <0.05, otherwise a random-effects model was used. Sensitivity analysis was performed to verify the robustness of the results by sequentially deleting single study and check the stability of the result. The underlying publication bias was visually examined by Begg's funnel plot and the degree of asymmetry was evaluated by Egger's test.

All the statistical tests were performed by STATA 14.2.

## Results

### Overall analysis

**Figure 1:**
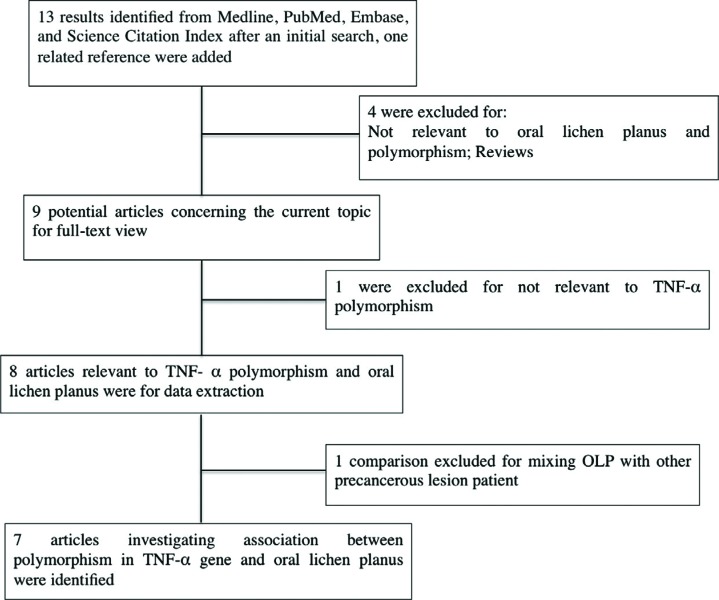
Flow chart for the process of selecting associated publications

**Figure 2:**
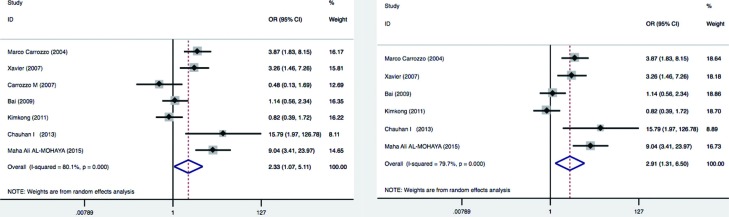
Summary of meta-analysis results. Left panel: Meta-analysis with a random-effects model for the association between OLP risk and the TNFα –308 G/A polymorphism (AA+AG vs. GG). Total random effects p= 0.03. Right panel: Meta-analysis excluding the HCV+ population study. Total random effects p=0.009. For each study, the estimate of OR and its 95%CI is plotted with a box and a horizontal line

**Figure 3:**
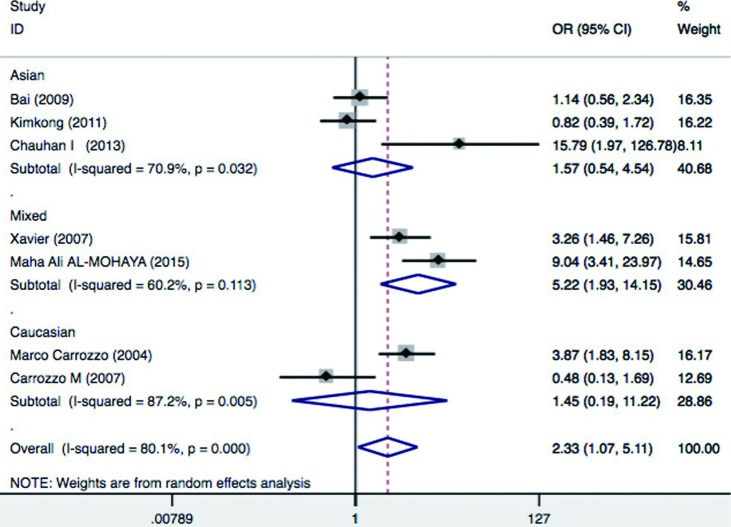
Forest plot of oral lichen planus (OLP) risk associated with TNF-α–308 G/A stratified by ethnicity. For each study, the estimate of OR and its 95%CI is plotted with a box and a horizontal line

The combined search yielded seven case-control studies that provided information on the association of OLP with TNFα –308 G/A polymorphism (Figure 1). In each studies the clinical diagnosis was confirmed histologically based on the WHO diagnostic criteria of oral lichen planus^19^. Three studies were from Asia^2^ ^,^ ^7^ ^,^ ^10^. Two were conducted in Italy^5^ ^,^ ^6^, one in Saudi Arabia^1^ and one in Brazil^19^. One study was specifically performed in patients with HCV positive infection^5^, two with negative HCV infection^2^ ^,^ ^6^, and four with mixed (studied population with or without HCV infection or HCV information not mentioned) HCV infection status^1^ ^,^ ^7^ ^,^ ^10^ ^,^ ^19^. We evaluated 450 patients and 867 controls. In each study, we tested for HWE using the *χ*2 test. The genotype distribution of the TNFα –308 G/A polymorphism among cases and controls in each study did not deviate from the expected HWE, except for the study conducted in Saudi Arabia^1^. Summary results of comparisons are listed in Figure 2. Distribution of genotypes are listed in Figure 3.

**Figure 4:**
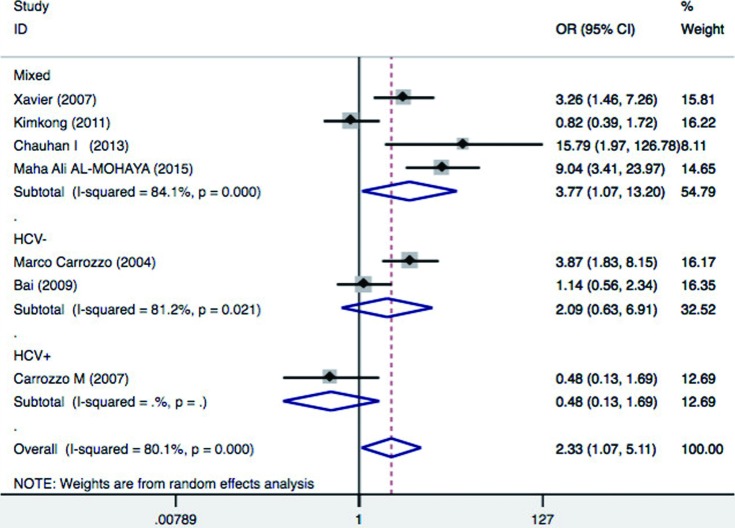
Forest plot of oral lichen planus (OLP) risk associated with TNF-α–308 G/A stratified by hepatitis C virus (HCV) infection status. For each study, the estimate of OR and its 95%CI is plotted with a box and a horizontal line

The heterogeneity for the seven studies included was analyzed. The TNFα –308 G/A polymorphism showed significant evidence of genetic heterogeneity between studies (*I* ^2^=80.1%, *p* = 0.03). Random-effects models should be used in the prescience of heterogeneity. Thus, the pooled effect was calculated with a random-effects model. In the pooled analysis, the TNFα –308 G/A polymorphism appeared to be a genetic risk factor for susceptibility to OLP with a random effects and odds ratio (OR) of 2.33 [95% confidence interval (CI): 1.07–5.11; *p* = 0.03 for AA+AG *vs.* GG model] (Figure 4, left panel). The results suggest that the AG heterozygote and AA homozygote had an increased risk of OLP compared with those individuals with the GG homozygote genotype. It appears that the effect of TNFα –308 G/A polymorphism may be confounded by HCV infection status in OLP^5^. Therefore, we considered HCV infection status and excluded one study that only studied Hepatitis C virus (HCV) infected OLP patients, and the meta-analysis yielded a higher significant association with a OR of 2.91 [95% confidence interval (CI): 1.31–6.5; *p*=0.009] (Figure 4, right panel).

### Subgroup analysis

**Figure 5:**
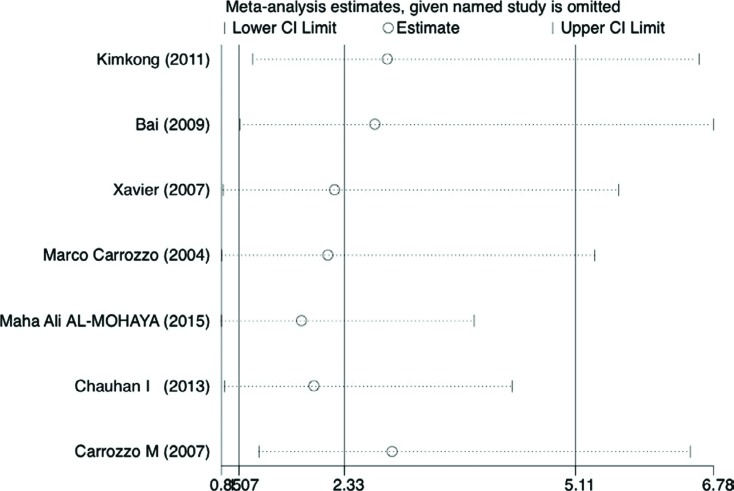
Sensitivity analyses. Effect of individual studies on the pooled OR under allele comparison for TNFα –308G/A polymorphism in OLP patients (random-effects model). Pooled odds ratios and 95% confidence intervals for excluding each data set in the meta-analysis. The vertical axis at 2.33 indicates the overall OR, and the two vertical axes at 1.07 and 5.11 indicate the 95% CI. Every hollow round indicates the pooled OR when the left study was omitted in a meta-analysis with a random model

The prevalence of OLP varies among different ethnic populations mainly due to variations in genetic background. Therefore, to determine whether the effects were due to ethnic differences, subgroup analysis by ethnicity was performed. As for ethnicity subgroup, our meta-analysis revealed that TNFα –308 G/A was associated with a significantly increased risk of OLP in mixed ethnicity (OR: 5.22; 95% CI = 1.93, 14.15 p=0.001), but not in Asians (OR=1.57; 95% CI = 0.54-4.54; *p*=0.41) and Caucasians (OR=1.45, 95%CI = 0.19-11.22, *p* = 0.72) for AA+GA *vs*. GG comparison (Figure 5). These results indicate that A allele carriers (AA+AG) had significantly higher risk of OLP in mixed ethnic population, but not in Asians and Caucasians.

**Figure 6:**
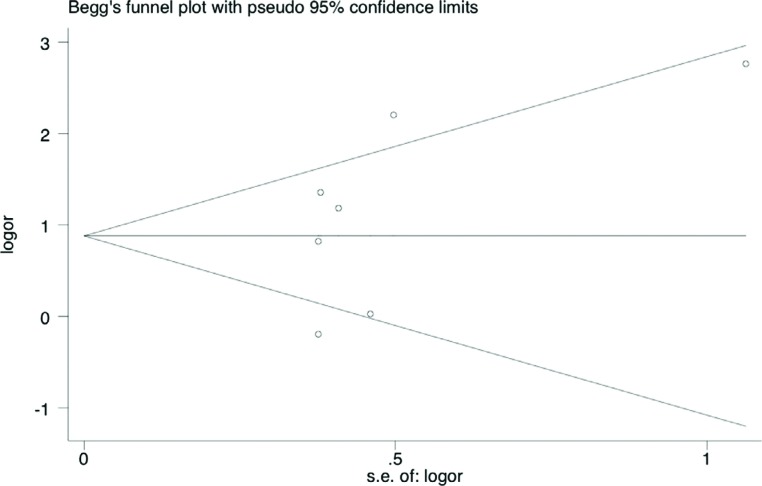
Begg's funnel plot for publication bias in selection of studies on the TNFα –308 G/A polymorphism. The horizontal line represents the meta-analysis summary estimate, and the diagonal linespseudo-95%CI limits about the effect estimate. In the absence of publication bias, studies will be distributed symmetrically above and below the horizontal line. Asymmetry on the bottom of the graph indicates evidence of publication bias towards studies. logOR, natural logarithm of the OR; s.e. of logOR, standard error of the logOR

Additionally, we performed subgroup analysis based on HCV infection status. Significant increased risk of OLP was found among patients with mixed HCV infection status (OR=3.77, 95%CI = 1.07–13.2, *p*=0.04), but not in patients without HCV infection (OR=2.09, 95%CI=0.63-6.91, *p*=0.225) and patients with HCV infection (OR=0.48, 95%CI = 0.13-1.69, *p*=0.25) (Figure 6). This suggest that A allele carriers (AA+AG) have significantly increased risk of OLP in patients with mixed HCV infection status, but not in HCV positive and/or negative patients. HCV infection also induce a heightened TNFα production, and thus may be a confounding factor in association studies between OLP risk and cytokine gene polymorphisms, underlying the need for future studies to investigate the HCV status of OLP patients. Aggregated ORs for the association are shown in Table 1 and the forest plots of OLP associated with TNFα –308 G/A (AA+AG *vs.* GG) in Figures 3, 4, 5 and 6.

**Table 1:** Associations between TNFα –308 G/A polymorphism and oral lichen planus (OLP)

Variables	n^a^	OR(95%CI)	P^b^
Overall	7	2.33 (1.07- 5.11) c	0.03
Overall excluding pure HCV+ population	6	2.91(1.30- 6.49) c	0.009
Subgroup by ethnicity			
Asian	3	1.57(0.54- 4.54) c	0.41
Caucasian	2	1.44(0.18-11.22) c	0.72
Mixed	2	5.22(1.92-14.15) c	0.001
Subgroup by HCV infection			
HCV+	1	0.47 (0.13- 1.69) c	0.25
HCV-	2	2.09(0.63-6.90) c	0.23
Mixed	4	3.77(1.07-13.19) c	0.04

a Number of comparisons.

b p value for Z-test.

c Random-effects model was used for all analysis

### Sensitivity analysis and publication bias

**Figure 7:**
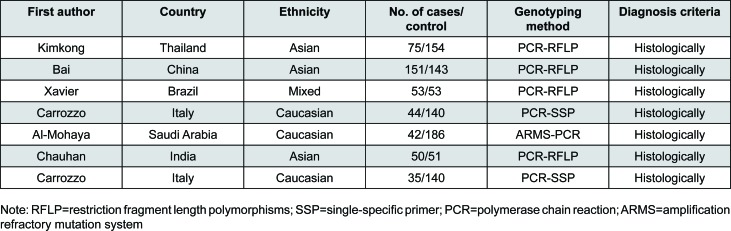
Characteristics of the seven case-control studies included. RFLP: restriction fragment length polymorphisms; SSP: single-specific primer, PCR: polymerase chain reaction; ARMS: Amplification Refractory Mutation System

In addition, sensitivity analysis was performed by removing one data set at a time. Statistically excluding any individual study did not resolve the genetic heterogeneity. The pooled OR was recalculated in the absence of each study, and OR was not significantly changed (Figure 7 and Table 2).

**Table 2:** Summary results of the sensitivity analysis

First author	Year	Country	OR	95% CI	p-value	I2 (%)
Omitting Kimkong, et al.	2011	Thailand	2,852	[1.2318 6.6029]	0.014	0.778
Omitting Bai, et al.	2009	China	2,7051	[1.0791 6.7810]	0.034	0.813
Omitting Xavier, et al.	2007	Brazil	2,2216	[0.8755 5.6376]	0.093	0.826
Omitting Carrozzo, et al.	2004	Italy	2,1411	[0.8577 5.3451]	0.103	0.814
Omitting Al-Mohaya, et al.	2015	Saudi Arabia	1,8232	[0.8529 3.8974]	0.001	0.755
Omitting Chauhan, et al.	2013	India	1,9743	[0.8949 4.3557]	0.092	0.811
Omitting Carrozzo, et al.	2007	Italy	2,9121	[1.3055 6.4961]	0.009	0.797

**Figure 8:**
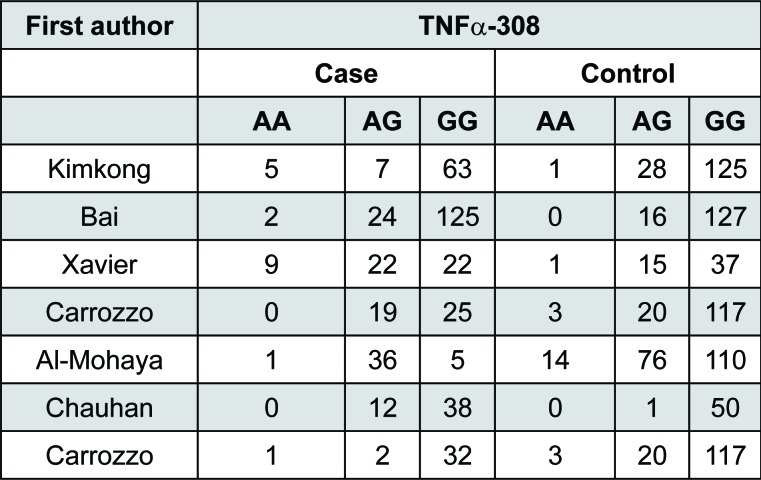
Distribution of TNF-α –308 genotype

The shape of the funnel plots did not reveal any evidence of obvious asymmetry (Figure 8), suggesting that there was no obvious publication bias. Egger's test showed no significant publication bias in this meta-analysis (t=0.65, *p*=0.54 for AA+AG *vs.* GG).

## Discussion

OLP is a complex disorder influenced by multiple genes and environmental factors. Genetic factors that influence the host immune response have been suggested to contribute to OLP risk^4^ ^,^ ^9^. The gene polymorphisms of TNFα has been reported to affect the susceptibility and the progression of OLP^2^ ^,^ ^10^. In this study, we focused on a functional polymorphism of TNFα –308 G/A. Based on seven case-control studies, our meta-analysis revealed that TNFα –308 G/A polymorphism was associated with a significant increase in the risk of OLP (OR=2.33; 95%CI:1.07-5.11; *p*=0.03). Hepatitis C virus has been proposed to be involved in the etiopathogenesis of OLP. Significant increased risk of OLP was found among patients with mixed HCV infection status (OR=3.77, 95%CI = 1.07-13.2, *p*=0.04), but not in patients without HCV infection (OR=2.09, 95%CI = 0.63-6.91, *p*=0.22) and patients with HCV infection (OR=0.47, 95%CI = 0.13-1.69, *p*=0.25). HCV infection induces an abnormal immune response in the host, enhancing TNFα production, and potentially influences the pathogenesis of OLP lesions. Thus, distinct pathogenetic mechanisms are involved in OLP patients with HCV infection and without HCV infection. The HCV status may be a confounding factor in association studies between OLP risk and TNFα gene polymorphisms, underlying the need for future studies to investigate the HCV status of OLP patients. When we excluded one research that only studied HCV infected OLP patients, the overall meta-analysis yielded higher significance of association with random effects OR of 2.91 [95% confidence interval (CI)=1.31-6.5; *p*=0.009].

Subgroup analysis based on ethnicity revealed that A allele carriers (AA+AG) had significantly higher risk of OLP in mixed ethnic population, but not in Asians and Caucasians. The two studies with mixed populations are from Brazil and Saudi Arabia respectively. Considering their complex racial genetic makeup, it may be one of the ethnic groups that account for the increased risk. Interestingly, both countries may have mixture of African ethnicity. This also justifies future studies focusing on African populations.

The current meta-analysis has several limitations. First, the sample size for some subgroup analysis was limited. Second, obvious heterogeneity was found in overall analysis. Moreover, one of the studies included in our meta-analysis did not follow HWE. Deviation from HWE reflects potential mistakes, such as laboratory or genotyping errors, population stratification or selection bias in controls. When we excluded one study that deviated from HWE, the pooled ORs was altered and the association became statistically insignificant (p>0.05). Lastly, in this study, we only evaluated a dominant model (AA+GA *vs.* GG). Other models [i.e. recessive model (AA *vs.* GG+GA) and homozygote model (AA *vs.* GG)] could have been conducted. Despite those limitations listed aforementioned, our meta-analysis also has its advantages. First, it contains the latest data on the association between TNFα –308 G/A polymorphism and OLP risk. Also, to our knowledge, it is the first meta-analysis that investigated the association between TNFα –308 G/A polymorphism and oral lichen planus risk.

## Conclusion

Our meta-analysis suggests that the substitution of G to A of TNFα –308 G/A polymorphism is a risk factor for OLP. Its effect exists among mixed populations with mixed HCV status. The TNFα –308 G/A polymorphism may be a useful genomic marker for oral lichen planus.
